# Innovative catalyst design for the oxygen reduction reaction for fuel cells

**DOI:** 10.1039/c6sc00139d

**Published:** 2016-02-11

**Authors:** Kenichi Shimizu, Lior Sepunaru, Richard G. Compton

**Affiliations:** a Physical and Theoretical Chemistry Laboratory , Department of Chemistry , The University of Oxford , South Parks Road , Oxford , OX1 3QZ , UK . Email: Richard.Compton@chem.ox.ac.uk ; Fax: +44 (0)1865 275 410 ; Tel: +44 (0)1865 275 413 ; Tel: +44 (0)1865 275 957

## Abstract

A combination of chemical and electrochemical catalysis is introduced herein as a new approach to overcome one of the most challenging and persistent issues in fuel cell cathodes. Demonstrated using hematite (α-Fe_2_O_3_) nanoparticles modified glassy carbon electrode, this bifunctional fuel cell catalyst system prevails the slow kinetics of the oxygen reduction reaction by rapid heterogeneous disproportionation of hydrogen peroxide. Whilst the catalytic efficiency of glassy carbon is limited to the two-electron reduction of oxygen, modification with hematite drastically improves it to equivalent to the four-electron pathway. This is due to regeneration of the cathodic fuel through the rapid decomposition of hydrogen peroxide. The importance of such system is stressed as the formation of water rather than hydrogen peroxide is essential to maximize the energy output of the fuel cell. Cycling of oxygen reduction/regeneration boosts the activity of a low-cost catalyst to be comparable to that of platinum and concurrently reduces the risk of cell degradation.

## Abstract 2

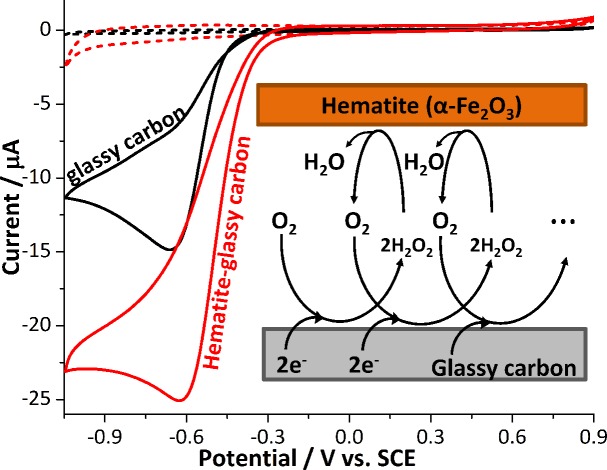
A bifunctional fuel cell catalyst system demonstrated herein overcomes the slow kinetics of the oxygen reduction reaction by rapid heterogeneous disproportionation of hydrogen peroxide.

## Introduction

Global warming caused by an excess greenhouse gas emission is an international concern. In need of reducing the global temperature incline below 1.5 °C per annum to comply with the Paris agreement,^1^ it is essential to develop clean and sustainable energy technology that is universally accessible. A fuel cell is one of the promising renewable energy devices that can be distributed to different parts of the world regardless of the weather conditions or the application. Oxygen is considered as the best cathodic fuel because of its high redox potential to from water (*E*^0^ = 1.229 – 0.059 pH V *vs.* NHE), virtually unlimited abundance in the Earth's atmosphere, and no emission of pollutants. The slow reaction kinetics is, however, a major limitation of the fuel cell performance, and platinum has been used extensively as a cathode catalyst. Today, distribution of this technology is hindered partly by the use of this noble metal catalyst as it is scarce in nature and expensive. Therefore, notwithstanding the countless efforts/progress made thus far,^2^ finding an affordable cathode catalyst is an urgent task.

The oxygen reduction reaction ideally takes the four-electron pathway (eqn (1)) to ensure that the fuel cell generates the maximum power output and water as the only exhaust. Alternatively, it can undergo the two-electron pathway forming hydrogen peroxide (eqn (2), *E*^0^ = 0.695 – 0.059 pH V *vs.* NHE) because of a number of reasons including insufficient catalytic activity.

1 O_2_ + 4H^+^ + 4e^–^ → 2H_2_O

2 O_2_ + 2H^+^ + 2e^–^ → H_2_O_2_

The lesser number of electrons involved in the cathodic process in eqn (2) seriously compromises the energy yield of the fuel cell. For example, for a H_2_/O_2_ fuel cell, 
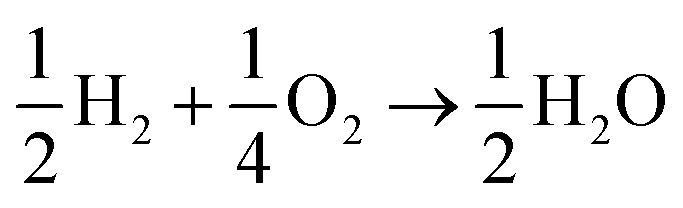
, has Δ*G*^0^ of –118.6 kJ mol^–1^ as compared to 
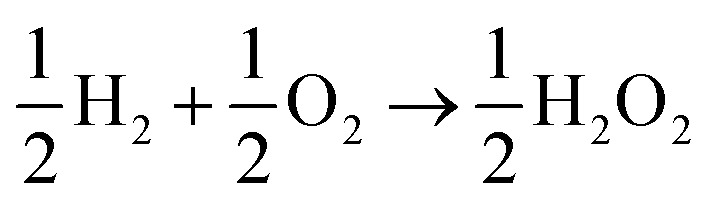
, which has Δ*G*^0^ of –67.1 kJ mol^–1^.^3^ In addition, a presence of an excess hydrogen peroxide can lead to a formation of peroxide radicals *via* a disproportionation reaction, which could impair cell membranes and other supporting materials.^4^–^6^ In order to avoid the fuel cell performance to be limited by eqn (2), hydrogen peroxide, if formed, must be quickly reduced to water, as in eqn (3), before it escapes into the bulk solution.

3 H_2_O_2ads_ +2H^+^ + 2e^–^ → 2H_2_O

The subscript indicates that the molecule usually has to be adsorbed onto the catalyst surface in order for the above reaction to take place.^2^,^7^ The reaction mechanism summarised by eqn (2) and (3) occurring in series is referred to as the 2 × 2 pathway, which give the catalysis as much efficiency as the four-electron pathway.^7^,^8^ This mechanism is however not applicable to a low-cost catalyst such as carbon in the fuel cells because eqn (3) takes place at a much more negative potential than eqn (2) and is energetically unfavorable.^9^

In this paper, we propose that electrochemically generated hydrogen peroxide can be converted to water through heterogeneous decomposition (eqn (4)).^10^,^11^

4 2H_2_O_2_ → O_2_ + 2H_2_O

Application of this mechanism to fuel cells could be beneficial as the cathodic fuel can be reproduced on site. Herein, hematite (α-Fe_2_O_3_) is utilized as a chemical catalyst for decomposing hydrogen peroxide to facilitate oxygen reduction reaction at a glassy carbon electrode. Hematite is an attractive material for alternative energy devices owing to its thermodynamic stability, low cost, facile synthesis, and natural abundance.^12^ Moreover, this mineral has been shown to have sufficient catalytic property for this purpose.^13^–^15^ Carbon has advantages over other electrocatalysts owing to its stability in neutral solution, low price, natural abundance, versatilely, electrical connectivity, and light weight.

The catalytic performance of the hematite-glassy carbon system is demonstrated in a neutral chloride solution. This is because there is an increasing interest in biological and enzymatic fuel cells, which are operated at around physiological pH and a highly efficient inorganic cathode catalyst can improve the cell performance. In addition, devices that are operated at a condition near the natural and physiological settings using the materials that are abundant or have a renewable source are environmentally and economically more sustainable for wider distribution of this green technology. The practical advantage of using a chloride solution is that it is one of the most common anions present in surface waters. Chloride ion reportedly affects the performance of precious metal catalysts, notably platinum, by blocking oxygen adsorption onto the catalyst surface.^16^,^17^ This is not the case for the hematite–carbon catalyst system described in this work. The successful application of this bifunctional catalyst system demonstrated herein introduces a new way of designing a cost-effective cathode catalyst.

## Results and discussion

### Catalytic reduction of oxygen

**Fig. 1:**
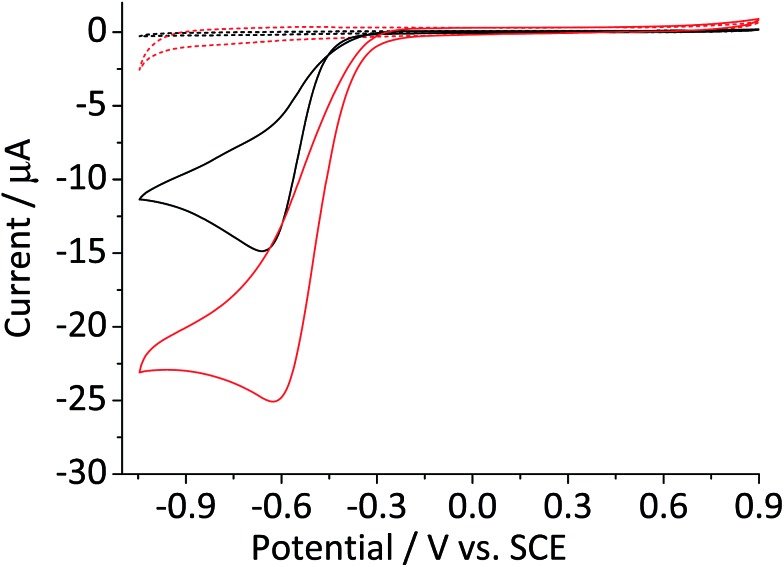
Cyclic voltammograms of the oxygen reduction reaction at a bare glassy carbon electrode (black) and that modified with 2.4 × 10^–5^ g cm^–2^ of hematite nanoparticles (red) collected in O_2_ saturated 20 mM KCl at the scan rate of 0.01 V s^–1^. The results of the two electrodes in the O_2_-free condition are also indicated with dashed lines of the same color. Potential is reported against a Standard Calomel Electrode (saturated KCl, SCE) throughout this article.

Fig. 1 compares the cyclic voltammetric responses of a bare glassy carbon electrode and that modified with 2.4 × 10^–5^ g cm^–2^ of hematite nanoparticles (equivalent to an average of 1.5 monolayers on the surface) in an O_2_ saturated 20 mM KCl solution. The voltammetric curve of a bare working electrode (black line) illustrates the 2-electron transfer oxygen reduction reaction, which is characteristic of the activity of glassy carbon.^10^ In comparison, there is a marked increase in the voltammetric performance of the modified electrode. This is not attributable to electro-catalytic activity of hematite, as this mineral is an n-type semiconductor and is not electronically conductive under the experimental conditions of the present study. This result therefore shows that the mineral nanoparticles are able to boost the electrocatalytic process taking place at a glassy carbon electrode. An excellent enhancement in the oxygen reduction reaction can also be seen from the significant anodic shift in potentials at which the current reaches 1 μA; at –0.31 V and –0.43 V *vs.* standard calomel electrode (saturated KCl, SCE) for modified and the unmodified electrode, respectively. On the other hand, the peak potentials do not differ significantly between the two electrodes. This is indicative of that the catalytic activity of hematite is independent of electrode potential.

**Fig. 2:**
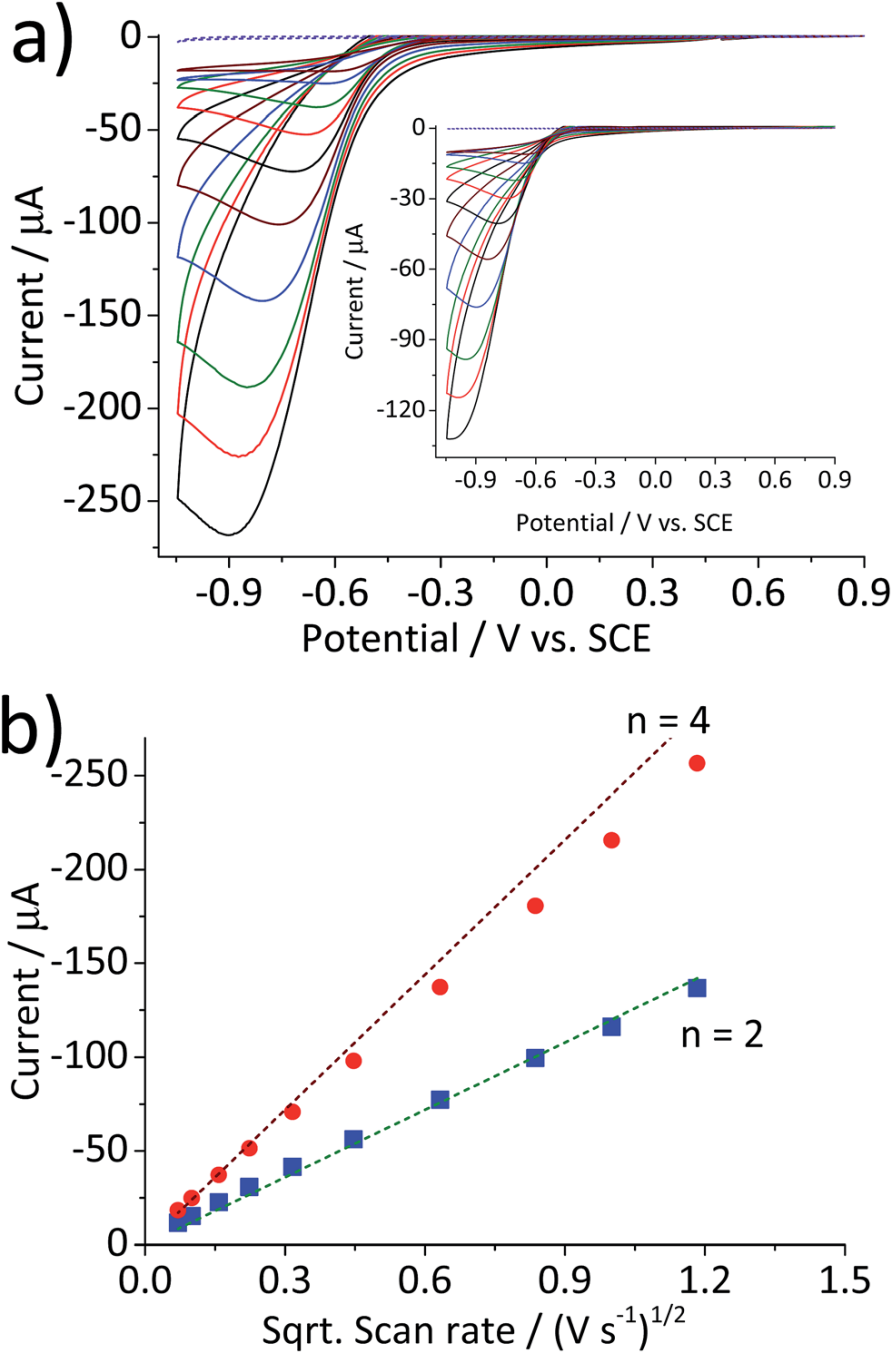
(a) Cyclic voltammograms of a glassy carbon electrode modified with 2.4 × 10^–5^ g cm^–2^ of hematite nanoparticles in O_2_ saturated 20 mM KCl at the scan rate varied from 1.4 V s^–1^ to 0.005 V s^–1^. (b) Plots of peak current as a function of the square root of the scan rate for the hematite modified electrode (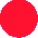) and for the bare electrode (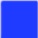). Dashed lines are calculated using eqn (5) for the 2-electron and the 4-electron reactions.

Scan rate dependency of the cyclic voltammetric response of the hematite modified electrode was examined by varying the scan rate from 1.4 V s^–1^ to 0.005 V s^–1^. The resulting cyclic voltammograms in Fig. 2a illustrate an increase in the peak current with respect to the scan rate. A similar result with a lower current output was observed from a bare glassy carbon electrode (Fig. 2a inset). For an electrochemical process that is controlled by the diffusion of redox active molecule, the peak current is directly proportional to the square root of scan rates, as shown in Fig. 2b. Furthermore, for fully irreversible process such as oxygen reduction reaction in this study, the correlation can be expressed by the Randles–Ševčík equation:^18^–^20^

5 *I* = 2.99 × 10^5^*n*(*n*′ + *α*)^1/2^*AD*^1/2^*Cν*^1/2^

where *n* is the number of electrons in an overall electrochemical process, *n*′ is the number of electrons involved prior to the rate determining step, *α* is the transfer coefficient, *A* is the geometrical surface area of working electrode, *D* is the diffusion coefficient of oxygen, *C* is a concentration of oxygen in the bulk solution, and *ν* is scan rate. Our result is in an excellent agreement with a theoretical plot generated from eqn (1) using previously reported values for *D* (=1.95 × 10^–5^ cm^2^ s^–1^),^21^*C* (=1.26 × 10^–6^ mol cm^–3^),^22^*n*′ (=0)^23^ and *α* (=0.26),^23^ and *n* = 2 for the reduction of oxygen to hydrogen peroxide, which is known to take place at a glassy carbon electrode.^24^ When the above constants are applied for the result obtained from the hematite modified electrode, an *n* value of 3.7 is extracted. Considering that the four-electron pathway is the most efficient catalytic process for oxygen reduction and essential to get maximum energy output of the fuel cell, our catalyst is highly comparable to conventional fuel cell catalyst such as platinum. Furthermore, our value is in reasonable agreement with values of 3.45 and 3.37 reported for hematite nanoparticles supported on carbon nanotubes.^25^,^26^

### Catalytic decomposition of hydrogen peroxide

**Fig. 3:**
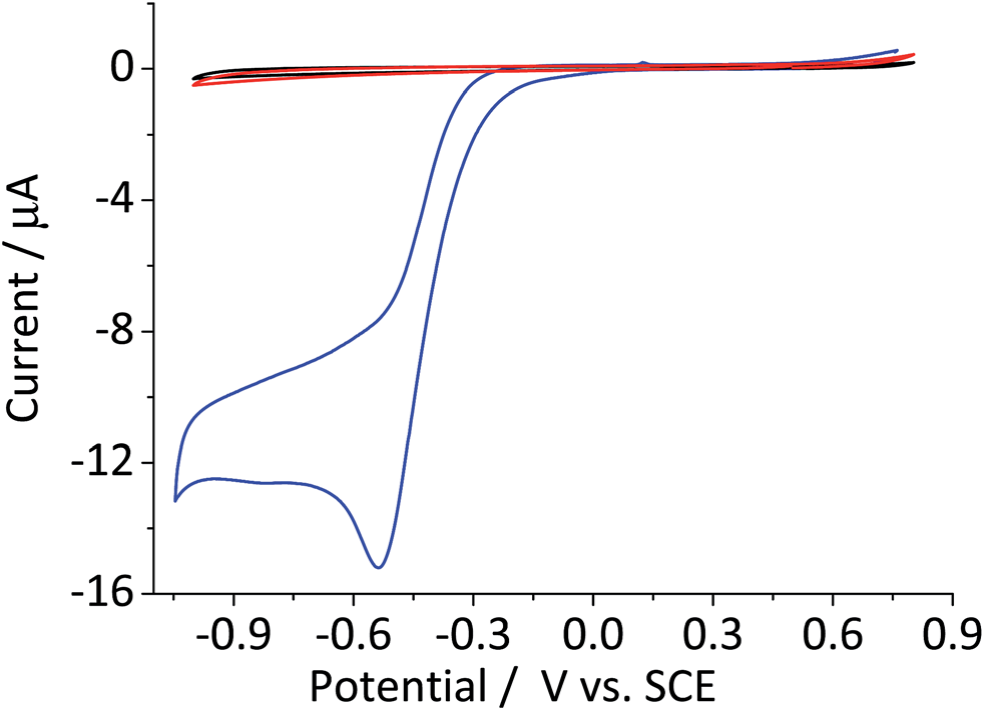
Cyclic voltammetric curves of a bare glassy carbon electrode (red) and that modified with 2.4 × 10^–5^ g cm^–2^ of hematite nanoparticles in N_2_ saturated 20 mM KCl with 1.5 mM H_2_O_2_ collected at the scan rate of 0.01 V s^–1^. Black line illustrates a cyclic voltammetric curve of bare glassy carbon in the same KCl solution without H_2_O_2_.

The catalytic decomposition of hydrogen peroxide was first reported by Broughton *et al.*^27^ using manganese(iv) dioxide. It was further examined by Hart *et al.*^13^ who studied the catalytic activity of various other oxides including cobalt(ii) and (ii) oxides and hematite. The catalytic decomposition of hydrogen peroxide (eqn (4)) at metal oxide surfaces typically follows first order kinetics with respect to the concentration of hydrogen peroxide.^13^,^14^ Fig. 3 shows the cyclic voltammogram of a hematite modified glassy carbon electrode that was recorded in a deaerated 20 mM KCl solution with 1.5 mM hydrogen peroxide. The difference in modified and unmodified glassy carbon electrode is obvious in the figure. Electrochemical reduction of hydrogen peroxide does not readily take place at glassy carbon electrode as illustrated in the figure (Fig. 3, red curve). Whereas, the voltammetric wave for the modified electrode generated substantially larger current output (Fig. 3, blue curve). The magnitude of the peak current is consistent with the decomposition of hydrogen peroxide to oxygen followed by electrochemical reduction of the product molecule. This leads to the hydrogen peroxide molecule generated from electrochemical reduction of oxygen to be decomposed repeatedly at the modified electrode surface. Such a cycle of decomposition/regeneration likely contributes to the catalytic performance of the hematite-glassy carbon bifunctional material. Moreover, this mechanism is probably attributable to the enhanced catalytic performance seen in a number of previous studies that employed various metal oxides including that of manganese,^28^–^30^ cobalt,^30^,^31^ and iron^25^,^26^,^32^ as a fuel cell cathode catalyst or a co-catalyst.

### Efficiency of the bifunctional catalyst

**Fig. 4:**
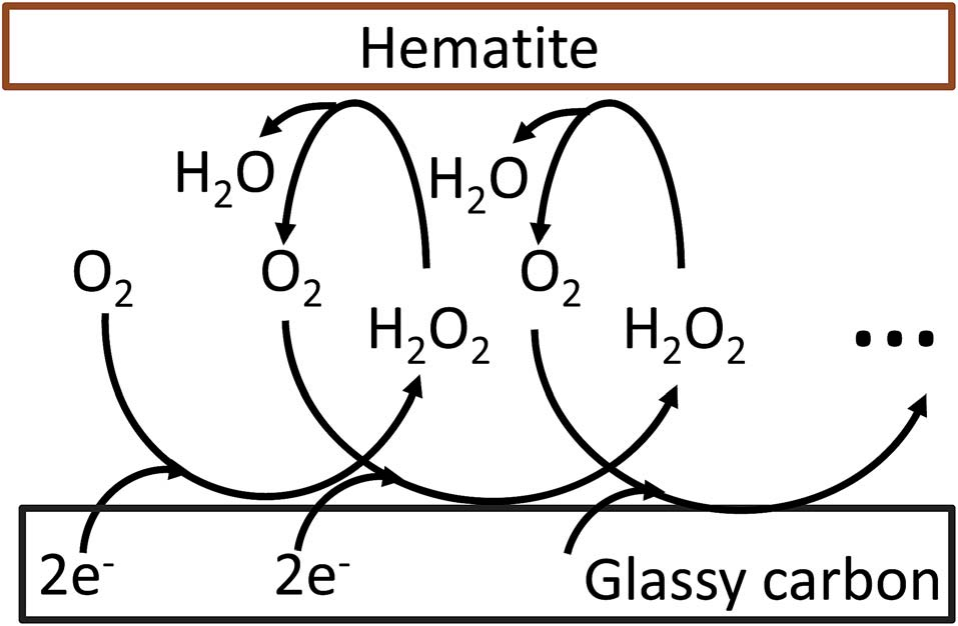
Graphical representation of the oxygen reduction/regeneration cycle at the hematite modified glassy carbon electrode.

The oxygen reduction reaction typically follows the first order kinetics with respect to the concentration of oxygen at the electrode surface.^10^ Therefore, the current output (and the efficiency of a catalyst) depends directly on the concentration of oxygen at the electrode surface. When the rate of hydrogen peroxide decomposition is much faster than the electrochemical process, total oxygen concentration at the modified electrode surface arises from the bulk diffusion and the on-site regeneration. This concept is visualized in Fig. 4. As a half of the oxygen concentration is chemically recovered after each cycle, surface concentration of oxygen can be expressed with respect to the frequency of the oxygen reduction/regeneration cycle:6
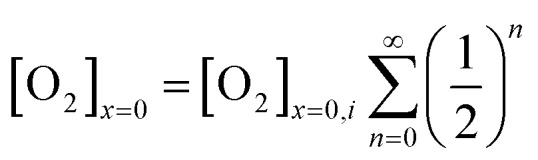
where [O_2_]_*x*=0_ is the oxygen concentration at the electrode surface, the subscript ‘*i*’ indicates the initial concentration, and *n* is the number of cycles. The sigma notation is the amplification coefficient, indicating the contribution from a chemical catalysis. For large *n* value, the sigma function becomes equal to 2, and hence, the system has the maximum catalytic efficiency of the four-electron pathway. In this work, the amplification coefficient, which is the peak current ratio of the modified and unmodified electrodes, is consistently around 1.7 irrespective of the scan rate. This value is less than the maximum attainable by this system. The main reason for this is attributed to the modification technique used in this work, which hematite is not perfectly monodispersed on the electrode surface. Inhomogeneous surface coverage allows hydrogen peroxide to escape into the bulk solution. Nonetheless, it is shown that the key mechanism of the bifunctional catalyst system is effective confinement of oxygen within the modified electrode matrix. The efficiency of the bifunctional catalyst system can be further improved through optimization of surface coverage and choice of a chemical catalyst.

## Conclusions

This study introduces a new concept of a bifunctional catalyst system, which combines chemical and electrochemical reactions, for the oxygen reduction reaction. In this system, the addition of a chemical catalyst for the heterogeneous disproportionation of hydrogen peroxide enhances the current output, as demonstrated using a hematite modified glassy carbon electrode. It is highlighted that the mixture of chemical and electrochemical catalysts attains the maximum catalytic efficiency of the four-electron pathway. From a mechanistic aspect, high catalytic efficiency is achieved through repeated cycling of the oxygen reduction/regeneration at the electrode surface. A critical importance of this work is that the low-performance electrocatalyst can be made as efficient as platinum when there is a supporting catalyst for *in situ* heterogeneous decomposition of hydrogen peroxide. Furthermore, this innovative catalyst system could lower the cost by utilizing cheaper raw materials, and the use of a chemical catalyst can also contribute to extending fuel cell lifetime by preventing cell degradation due to peroxy radicals.

## Experimental

### Materials

All chemicals were used as received. Ferric chloride was purchased from Merck (pro analysi), and hydrochloric acid was obtained from VWR (analytical grade). Potassium chloride (≥99.0%) and hydrogen peroxide (∼30%) were purchased from Sigma Aldrich. Ultrapure water used in this work was filtered through Millipore SimPak® 1 purification pack (lot# F5BA50456). Oxygen free N_2_ gas (99.998%, BOC Gases plc, Guildford, UK) and O_2_ gas (99.5%) were humidified by passing through traps containing ultrapure water and *ca.* 0.1 M NaOH.

### Hematite synthesis and characterization

Hematite nanoparticles were synthesized in accordance with ref. 33. Briefly, it was carried out by adding a 0.72 M FeCl_3_ solution dropwise to a preheated 3 mM HCl solution while it was being vigorously stirred. Subsequently the mixture was placed in an oven at 100 °C for a week to allow the formation of nanoparticles. Unreacted ions are removed by dialysis with ultrapure water until the dialysate has the resistivity of at least 10^6^ Ω cm. The resulting nanoparticles were stored in a suspension in the dark. Transmission electron microscopic (TEM) image of the metal oxide nanoparticles was taken by a Jeol JEM 1230 microscope equipped with a digital multi-scan camera (Gata MSC 600CW). Particle shapes are in good agreement with previous reports for the mineral prepared in a similar manner.^34^,^35^ The mean diameter of 215 particles identified from the TEM images was 36.8 ± 6.6 nm. Powder X-ray diffraction spectroscopy was performed by a Bruker d8 Advance X-ray diffractometer (XRD) using Cu Kα radiation. The scanning angle was swept from 20° to 80° at a rate of 0.008° s^–1^, and the resulting XRD spectrum was compared. The X-ray diffraction pattern matches with the reference patterns of the rhombohedral structure (PDF#072-0469) of hematite.^36^,^37^ The crystal size suggested from the XRD diffractogram is 32.1 nm, which is in agreement with the TEM observation. X-ray photoelectron spectroscopic (XPS) analysis was conducted by a Kratos Axis Ultra electron spectrometer with a delay line detector and a monochromated Al Kα source operated at 150 W. Wide range XPS survey spectra (from 1100–0 eV at 160 eV pass energy) found no impurity other than the carbon-based impurity, which is commonly observed in XPS analyses and irrelevant to the sample purity.^38^ High-resolution spectra (collected at a pass energy of 20 eV) of Fe 2p exhibited a shoulder bump at 709.8 eV, a characteristic Fe 2p_3/2_ peak shape of hematite suggesting that formation of another iron oxy(hydro)oxides is negligible.^38^,^39^ The O 1s spectra showed predominantly O^2–^ at 529.9 eV originated from the structural oxygen, as well as peaks associated with non-stoichiometric surface OH groups at 531.2 eV and water residue at 532.1 eV.^33^,^40^–^42^ The (O + OH) to Fe atomic ratio is 1.4 that is in good agreement with the expected value of 1.5 from the empirical formula of this mineral (Fe_2_O_3_). All spectra were processed with the CasaXPS software and the binding energy scale was adjusted with respect to the C 1s line of aliphatic carbon, 285.0 eV. Peak fit was carried out using the Shirley background correction and a linear combination of Gauss–Lorentz functions at 70 : 30 ratio.

### Electrochemical analysis

Electrochemical measurements were carried out in a conventional three-electrode cell consisting of a 3 mm diameter glassy carbon working (Bioanalytical Systems Inc.), a standard calomel electrode (saturated KCl, ALS Co. Ltd.) reference, and a Pt wire auxiliary electrodes. Glassy carbon working electrode was cleaned prior to the experiment by polishing on aqueous slurries of 1, 0.3, and 0.05 μm alumina (Buehler) in descending order of the size for 1 to 3 min each. It was then sonicated briefly in ethanol/water solution in order to remove alumina residue from the surface. A clean glassy carbon electrode was modified with hematite by drop casting a 2 μL droplet of an aqueous suspension that contained 0.864 g L^–1^ of the mineral and dried under ambient conditions. Voltammetric response of the modified electrode was first checked in 20 mM KCl solution that was thoroughly degassed with N_2_ bubbling. For electro-reduction of oxygen, O_2_ gas was used instead. The hematite modified glassy carbon electrode was immersed in a N_2_ saturated 20 mM KCl solution for 10 min prior to voltammetric analysis. Cyclic voltammetry was carried out at 25 °C in the dark condition, and a potential range between –1 and 0.9 V *vs.* Ag/AgCl was applied at various scan rates. All electrochemical measurements were conducted using a Metrohm μAutolab II potentiostat (Utrecht, the Netherland) with Nova (v.1.11.2) as an operating interface.

## References

[cit1] United Nations Framework Convention on Climate Change, Adoption of the Paris agreement, Proposal by the President, Paris, United Nations, 2015, available from http://www.unfccc.int/resource/docs/2015/cop21/eng/l09.pdf.

[cit2] Ge X., Sumboja A., Wuu D., An T., Li B., Goh F. W. T., Hor T. S. A., Zong Y., Liu Z. (2015). ACS Catal..

[cit3] Petlicki J., van de Ven T. G. M. (1998). J. Chem. Soc., Faraday Trans..

[cit4] Kinumoto T., Inaba M., Nakayama Y., Ogata K., Umebayashi R., Tasaka A., Iriyama Y., Abe T., Ogumi Z. (2006). J. Power Sources.

[cit5] Ghassemzadeh L., Kreuer K.-D., Maier J., Müller K. (2010). J. Phys. Chem. C.

[cit6] Zhang S., Yuan X.-Z., Hin J. N. C., Wang H., Friedrich K. A., Schulze M. (2009). J. Power Sources.

[cit7] Hermann V., Dutriat D., Müller S., Comninellis C. (2000). Electrochim. Acta.

[cit8] Wiggins-Camacho J. D., Stevenson K. J. (2011). J. Phys. Chem. C.

[cit9] Nissim R., Compton R. G. (2013). Phys. Chem. Chem. Phys..

[cit10] Neumann C. C. M., Laborda E., Tschulik K., Ward K. R., Compton R. G. (2013). Nano Res..

[cit11] Hasnat M. A., Rahman M. M., Borhanuddin S. M., Siddiqua A., Bahadur N. M., Karim M. R. (2010). Catal. Commun..

[cit12] Gray H. B. (2009). Nat. Chem..

[cit13] Hart A. B., McFadyen J., Ross R. A. (1963). Trans. Faraday Soc..

[cit14] Huang H.-H., Lu M.-C., Chen J.-N. (2001). Water Res..

[cit15] Hermanek M., Zboril R., Medrik I., Pechousek J., Gregor C. (2007). J. Am. Chem. Soc..

[cit16] Schmidt T. J., Paulus U. A., Gasteiger H. A., Behm R. J. (2001). J. Electroanal. Chem..

[cit17] Katsounaros I., Meier J. C., Mayrhofer K. J. J. (2013). Electrochim. Acta.

[cit18] Bard A. J. and Faulkner L. R., Electrochemical Methods: Fundamentals and Applications, John Wiley & Sons, New York, 2nd edn, 2001.

[cit19] Guidelli R., Compton R. G., Feliu J. M., Gileadi E., Lipkowski J., Schmickler W., Trasatti S. (2014). Pure Appl. Chem..

[cit20] Guidelli R., Compton R. G., Feliu J. M., Gileadi E., Lipkowski J., Schmickler W., Trasatti S. (2014). Pure Appl. Chem..

[cit21] Ju L. K., Ho C. S. (1985). Biotechnol. Bioeng..

[cit22] Oxygen and Ozone: Solubility Data Series, ed. R. Battino, Elsevier Science, 2015.

[cit23] Nissim R., Compton R. G. (2014). ChemElectroChem.

[cit24] Sundberg K. M., Smyrl W. H., Atanasoska L., Atanasoski R. (1989). J. Electrochem. Soc..

[cit25] Sun M., Dong Y., Zhang G., Qu J., Li J. (2014). J. Mater. Chem. A.

[cit26] Sun M., Zhang G., Liu H., Liu Y., Li J. (2015). Science China Materials.

[cit27] Broughton D. B., Wentworth R. L. (1947). J. Am. Chem. Soc..

[cit28] Choe J. E., You J.-M., Yun M., Lee K., Ahmed M. S., Üstundağ Z., Jeon S. (2015). J. Nanosci. Nanotechnol..

[cit29] Roche I., Scott K. (2008). J. Appl. Electrochem..

[cit30] Poux T., Bonnefont A., Kéranguéven G., Tsirlina G. A., Savinova E. R. (2014). ChemPhysChem.

[cit31] Su Y., Zhu Y., Yang X., Shen J., Lu J., Zhang X., Chen J., Li C. (2013). Ind. Eng. Chem. Res..

[cit32] Wang C., Daimon H., Sun S. (2009). Nano Lett..

[cit33] Shimizu K., Shchukarev A., Boily J.-F. (2011). J. Phys. Chem. C.

[cit34] Guo H., Xu H., Barnard A. S. (2013). Energy Environ. Sci..

[cit35] Wang W., Howe J. Y., Gu B. (2008). J. Phys. Chem. C.

[cit36] Blake R. L., Hessevick R. E., Zoltai T., Finger L. W. (1966). Am. Mineral..

[cit37] Sadykov V. A., Isupova L. A., Tsybulya S. V., Cherepanova S. V., Litvak G. S., Burgina E. B., Kustova G. N., Kolomiichuk V. N., Ivanov V. P., Paukshtis E. A., Golovin A. V., Avvakumov E. G. (1996). J. Solid State Chem..

[cit38] Yamashita T., Hayes P. (2008). Appl. Surf. Sci..

[cit39] Boily J.-F., Shchukarev A. (2010). J. Phys. Chem. C.

[cit40] Shchukarev A., Boily J.-F. (2008). Surf. Interface Anal..

[cit41] Desai L. D., Pathan H. M., Min S., Jung K., Joo O. S. (2005). Appl. Surf. Sci..

[cit42] Ferretto L., Glisenti A. (2002). J. Mol. Catal. A: Chem..

